# Mucosal Resident Memory CD4 T Cells in Protection and Immunopathology

**DOI:** 10.3389/fimmu.2014.00331

**Published:** 2014-07-14

**Authors:** Damian Lanz Turner, Donna L. Farber

**Affiliations:** ^1^Columbia Center for Translational Immunology, Columbia University Medical Center, New York, NY, USA; ^2^Department of Medicine, Columbia University Medical Center, New York, NY, USA; ^3^Department of Surgery, Columbia University Medical Center, New York, NY, USA; ^4^Department of Microbiology and Immunology, Columbia University Medical Center, New York, NY, USA

**Keywords:** mucosal immunity, T-cell memory, intestine, lung, tissue homing

## Abstract

Tissue-resident memory T cells (TRM) comprise a newly defined subset, which comprises a major component of lymphocyte populations in diverse peripheral tissue sites, including mucosal tissues, barrier surfaces, and in other non-lymphoid and lymphoid sites in humans and mice. Many studies have focused on the role of CD8 TRM in protection; however, there is now accumulating evidence that CD4 TRM predominate in tissue sites, and are integral for *in situ* protective immunity, particularly in mucosal sites. New evidence suggests that mucosal CD4 TRM populations differentiate at tissue sites following the recruitment of effector T cells by local inflammation or infection. The resulting TRM populations are enriched in T-cell specificities associated with the inducing pathogen/antigen. This compartmentalization of memory T cells at specific tissue sites may provide an optimal design for future vaccination strategies. In addition, emerging evidence suggests that CD4 TRM may also play a role in immunoregulation and immunopathology, and therefore, targeting TRM may be a viable therapeutic approach to treat inflammatory diseases in mucosal sites. This review will summarize our current understanding of CD4 TRM in diverse tissues, with an emphasis on their role in protective immunity and the mechanisms by which these populations are established and maintained in diverse mucosal sites.

## Introduction

The anatomic complexity of vertebrates necessitates an immune defense system, which provides protection at diverse sites of pathogen encounter. Earlier views of the immune system as a circulating, surveilling defense force have been supplanted by more recent evidence that the immune response is both localized and adapted to specific anatomic compartments. For T lymphocytes, seminal work by Leo Lefrançois and colleagues first revealed that virus-specific CD8 T cells that were generated and maintained as long-lived memory T cells after infection could be maintained in multiple tissue sites throughout the body (1). Subsequent studies using parabiosis models provided early evidence that certain tissues such as intestines contained populations of memory CD8 T cells that did not readily circulate (2). In recent years, non-circulating populations of memory CD8 T cells have been identified in skin, lung, vaginal mucosa, brain, and even in lymphoid tissues (3–7), which are collectively referred to as “Tissue-resident” memory CD8 T cells (CD8 TRM) (8, 9). TRM are populations of clonally expanded memory T cells that permanently reside in peripheral tissues, are maintained independently of lymphoid and circulating memory T-cell populations, and have the ability to respond rapidly to re-exposure to cognate antigen.

While most studies in mouse models of infection have focused on memory CD8 T-cell generation and maintenance to virus infection, less is understood about memory CD4 T cells and their role in protection and in tissue-specific responses. In both mice and humans, CD4 T cells are the most abundant lymphocytes throughout the body; they predominate in lymphoid tissue and memory CD4 T cells also outnumber memory CD8 T cells in mucosal tissues and barrier surfaces (10–12). Tissue-resident CD4 TRM have been identified in the lung, skin, and mucosal surfaces, and function to direct protective responses and coordinate recruitment of immune cells to tissues sites (7, 12–15). In addition to protective responses, there is also potential in any *in situ* immune response for collateral tissue damage, resulting in immunopathology. Since tissue-specific inflammatory disease can be driven by CD4 T-cell responses, the contribution of tissue-resident memory T-cell responses in these contexts is important to consider. In this review, we will focus on the role of CD4 TRM in immune responses, both protective and pathogenic and discuss current research and models for their generation and maintenance.

## Anatomic Heterogeneity of Memory CD4 T Cells: Early Studies

The effectiveness of T-cell mediated immunity against pathogens is partly derived from the wide distribution throughout the body of a large repertoire of individual T-cell clones with the ability to recognize and mount an effector response to a large number of pathogen-associated antigenic signatures. Naïve T cells express chemokine receptors such as CCR7 and L-selectin (CD62L) that target their migration from circulation through lymphoid tissue. This circulatory pattern provides the greatest probability of encounter of naïve T cells with their cognate antigens, which are presented by mature antigen presenting cells (APC) that ferry antigen from peripheral tissue to lymph nodes. Upon activation by antigen, naïve cells clonally expand and acquire effector properties, and in the process, upregulate expression of integrins and chemokine receptors that direct migration and access to inflamed peripheral tissues. During the ongoing immune response, effector cells are thus present in both lymphoid organs and peripheral tissues. While the majority of these activated and effector T cells die after antigen clearance, a proportion persists and develops into long-lived memory T cells.

The identification of memory CD4 T-cell heterogeneity in humans and mice based on homing receptor expression 15 years ago provided the initial evidence that T-cell memory was anatomically diverse. In humans, heterogeneity in CCR7 expression was identified among CD45RO^+^ memory CD4 T cells in blood in a landmark study, which designated the CCR7^hi^ memory subset as central-memory (TCM) and the CCR7^lo^ memory subset as effector-memory (TEM) (16, 17). There were also early indications of memory T-cell heterogeneity in mice based on CD62L expression in antigen-specific memory CD4 T cells generated from virus infection or peptide-specific priming, giving rise to CD62L^lo^ and CD62L^hi^ memory subsets (18–20).

Anatomic heterogeneity of memory CD4 T cells was subsequently demonstrated in mouse models and some human studies. Jenkins and colleagues showed in whole mouse studies that memory CD4 T cells generated in response to peptide immunization were found in both lymphoid and non-lymphoid sites, including in lung, liver, intestines, and salivary glands (21). Other studies identified antigen-specific memory CD4 T cells in mouse lungs following respiratory virus infection (22), or from adoptive transfer of effector cells (23). Similarly, memory CD4 T cells were identified in mouse bone marrow (24), female reproductive tract (FRT) (25), and skin (26). Similarly, early studies in human tissue identified memory CD4 T cells in tonsils and non-lymphoid tissues isolated from surgical explants (27). Additional populations of human memory CD4 T cells were also identified in skin (28) and cerebrospinal fluid (29). These initial findings suggested that memory T cells may circulate through multiple and diverse sites. However, early evidence of phenotypic and functional distinction between memory CD4 T cells in tissues compared to those in spleen or circulation (23, 28), suggested that these tissue memory populations may be maintained independent of their counterparts in circulation.

Several new technological approaches were subsequently implemented to study whether memory T cells could take up residence and be retained in tissue sites as well as to distinguish circulating from tissue-resident memory T cells. Parabiosis experiments in which mouse pairs are surgically conjoined to create shared circulations provided direct evidence for memory CD4 T cells retained in lung tissues (13), and for memory CD8 T cells resident in intestines and skin (2, 3). Imaging via confocal or intravital microscopy also demonstrated that specific T cells are localized in niches within tissues (12, 30, 31). However, it is still difficult to assess whether immune cells isolated from peripheral tissues are present within microcapillaries of the tissues or are resident within the tissue. To overcome this problem, an increasing number of studies have used *in vivo* antibody labeling of T cells with a fluorescently labeled antibody prior to tissue harvest, such that T-cell accessible to circulation become labeled *in vivo* with antibody, while those within tissues and not in circulation are protected by *in vivo* labeling (6, 12, 13, 32). In response to infection, memory CD4 T cells that are protected from *in vivo* antibody labeling have been identified in lungs following respiratory infection with influenza virus, *Mycobacterium tuberculosis* (Mtb), and systemic infection with LCMV (12, 13, 15, 32). When combined with imaging approaches, both circulating and resident memory CD4 T cells can be identified in mouse lungs and spleen. In the following sections, we present the current state of knowledge about CD4 TRM in general and the specific role of CD4 TRM in mucosal sites.

## Resident Memory CD4 T Cells and Protective Immunity

### CD4 TRM: General properties

CD4 TRM are defined as non-circulating, memory CD4 T cells that are not readily accessible to the vasculature and are retained locally in specific tissue sites. Phenotypically, mouse CD4 TRM are distinguished from circulating TEM populations based on upregulated expression of the early activation marker CD69 and the integrin CD11a (12, 13, 33). CD69^+^ memory CD4 T cells have been identified in mouse lungs, skin, and intestine, while spleen contains only a minority proportion of CD69^+^ memory CD4 T cells (12, 13). In humans, CD4 TEM phenotype cells in lungs, intestines, lymph nodes, and bone marrow express CD69, with 50–60% of spleen CD4 TEM expressing CD69, while TEM circulating in blood uniformly lack CD69 expression (10, 34, 35). The specific upregulation of CD69 by tissue memory CD4 T cells suggests that memory CD4 T cells in human tissues perceive distinct signals compared to those circulating in blood (36). While CD8 TRM are also characterized by upregulation of the α_E_ integrin, CD103 (9), CD4 TRM in mucosal and lymphoid sites in human and mice generally do not express CD103 (12, 36), except for a proportion of skin memory CD4 T cells (31). Whether CD4 TRM in specific sites express other tissue-specific or TRM-specific integrins or adhesion markers is not known, although expression of the collagen-binding integrins VLA-1 and α2 are associated with lung effector CD4 T-cell responses and bone marrow memory CD4 T cells, respectively (37, 38). Functionally, CD4 TRM exhibit rapid recall function and can produce IFN-γ and IL-17 in mucosal sites, although the extent to which their functional profile differs from circulating memory populations is not well characterized.

These observations support the general concept that TRM are an effective first line of defense against invading pathogens due to their localization in mucosal tissues that are frequently the sites of infection. TRM populations are likely derived from clonally expanded populations of effector T cells responding to an infection, and therefore, contain relatively high frequencies of T-cell clones specific for pathogens that target individual tissue sites. This emerging hypothesis postulates that while TRM provide an immediate *in situ* immune response to infection, TCM and TEM located in lymphoid organs provide a delayed response due to their reliance on migration of APCs for the initiation of the response. The relative contribution of each component to conferring protective immunity will probably differ based on the tissue(s) that is infected and the nature of the pathogen; however, this is currently a major research focus. Our knowledge of CD4 TRM and their properties is quickly expanding and it is likely that they will be identified in additional tissues and implicated in immune protection against a variety of tissue-tropic pathogens. A summary of current observations of CD4 TRM in mucosal sites and their protective capacities in different pathogen models is presented in Table 1.

**Table 1:** **Observations of CD4 TRM in mucosal tissues**.

Tissue	Pathogen/antigen	Features	Reference
Lung	Influenza virus	CD69^+^, cluster around airways	(12, 13)
		Unaffected by FTY720 treatment	
		Lung-tropic, protect against second infection	
	Influenza virus (humans)	Virus-specific memory CD4 T cells enriched in lung, CD69^+^, VLA-1^+^	(34, 39)
	*Mycobacterium tuberculosis*	CD69^+^, CXCR3^hi^, PD-1^hi^, KLRG1^lo^, lung-tropic. Protect against second infection	(15)
	*Mycobacterium tuberculosis*	Lung CD4 TRM generated by BCG vaccination	(40)
		CD4 TRM enhances MHC II on lung macrophages during 2° challenge	
	*Nippostrongylus brasiliensis*	Pathogen-specific production of IL-4 and IL-13	(41)
		Lung TRM unaffected by FTY720 treatment	
		Protect against second infection	
Female genital tract	Herpes simplex virus (humans)	Enrichment of antigen-specific CD4 T-cell clones in cervical cytobrush specimens and genital lesions	(42, 43)
	Herpes simplex virus (mice)	CD4 TRM generated in vaginal mucosa (no CD8)	(44)
		IFN-γ-mediated protection against 2° HSV challenge	
Gut	*Listeria monocytogenes*	Primary and second oral infection *Listeria* generates long-lived antigen-specific T-cell population in LP	(45)
	N/A	Homeostatic proliferation of naïve CD4 T cells in MLN generates gut-tropic, α_4_β_7_^+^, T_H_17 cells	(46)

### Lung CD4 TRM

The lung or respiratory tract is a major site for entry of viral and bacterial pathogens, with respiratory infections constituting the most prevalent cause of illness globally and throughout an individual’s lifetime. It has been known for some time that respiratory viral infections induce TEM populations within the lung that display an activated phenotype (47), and that these populations persist within the lung tissue and the lung airways following infection (22). Due to the possible inclusion of cells within the microcapillaries of the lung, these previous studies found phenotypic heterogeneity among lung memory CD4 T-cell isolated from digested tissue (48). Introduction of virus-specific memory CD4 T cells directly into the respiratory tract by intranasal delivery, provided protection to secondary virus challenge (22); however, it was not established whether these protective subsets were circulating or remained resident in lung tissue.

CD4 TRM in the lung were the first resident memory CD4 T-cell population to be extensively characterized and demonstrated to exhibit protective function. Using the *in vivo* labeling technique to analyze lung memory T-cell populations following influenza virus infection, we found that CD4 TRM were phenotypically distinct from circulating TEM populations in their expression of high levels of CD69 and CD11a, and in their residence in a distinct niche of the lung near airways (12). Further evidence of distinct properties of lung effector-memory T cells come from adoptive transfer and parabiosis experiments. These studies showed that lung memory CD4 T cells specifically migrate back to the lung following adoptive transfer into congenic hosts while spleen-derived memory CD4 T cells migrate into multiple tissues (13). Parabiosis further revealed that lung memory CD4 T cells were specifically retained in lungs while spleen-derived memory CD4 T cells freely recirculated among multiple lymphoid tissues and entered the lung, but were not retained there (13). Moreover, lung CD4 TRM generated following influenza infection were maintained longterm and were unperturbed in the presence of inhibitors of lymphoid egress and inducers of lymphopenia (12). Similarly, Mtb infection in mice resulted in generation of lung-tropic and retentive CD4 TRM as well as circulating TEM cells (15). Moreover, human memory CD4 T cells in lung are predominantly a TEM phenotype with upregulated expression of CD69 (10, 34). Together, these studies identified a new subset of lung CD4 TRM with distinct phenotypic, migration, retention, and maintenance properties.

In experimental models of respiratory infection with influenza, parainfluenza virus, and Mtb, the resulting lung TRM population is enriched with pathogen-specific CD4 (12, 15, 49) and CD8 T cells (12). Likewise, the lungs of human subjects that had been exposed to Mtb contain resident memory CD4 T cells that were specific for Mtb antigens (50). CD8 T cells specific for influenza and respiratory syncytial virus are found in higher frequencies within human lungs than in the spleen, blood, and skin (12, 34, 39). While it is possible to determine Mtb exposure by a PPD skin test, it is difficult to document the history of influenza and parainfluenza virus infection in human subjects. The high prevalence of IAV infection among the population, however, suggests that the compartmentalization of IAV-specific T cells within the lung is likely a consequence of local infection.

The elevated precursor frequency of pathogen-specific cells in the lung is thought to direct an early *in situ* immune response against secondary infection. In support of this hypothesis, it has been reported that there is local activation and expansion of memory CD4 T cells in the lung upon secondary IAV challenge (49). We have likewise found that lung CD4 TRM can produce effector cytokines at early time points following secondary viral infection (33). Rapid recall of memory CD4 T cells in the lung has also been suggested as being integral for protection against Mtb in both mouse and human studies (50–52). Lung CD4 TRM in mice were found to mediate superior protective responses to influenza virus challenge compared to spleen-derived memory CD4 T cells (13). Interestingly, influenza-specific lung CD4 TRM protected from morbidity of infection while also mediating rapid viral clearance, and carried out these functions *in situ* without extensive proliferative expansion or migration to other sites (13). In a mouse Mtb infection model, CD4 TRM cells conferred better protection from secondary Mtb infection in susceptible hosts than their circulating intravascular counterparts (15). The mechanisms for protection by CD4 TRM in the lung have not yet been elucidated. While IFN-γ is important for memory CD4 T-cell-mediated recall responses to influenza (53, 54), protection for Mtb was not associated with IFN-γ production (15).

In humans, protection due to resident T cells is difficult to assess. One group has used the novel approach of bronchoscopic antigen challenge with purified protein derivative of Mtb (PPD) to assess the role that local lung memory T cells play in the secondary immune response to Mtb infection. By comparing the local lung immune response (after bronchoscopic challenge) of healthy individuals with a positive PPD skin test to healthy PPD negative controls they observed rapid mobilization of CD4 T cells into the lung airways (48 h) resulting in a significant increase in antigen-specific T cells (55). These early responding cells did not undergo proliferative expansion as assessed by Ki67 staining, suggesting that they may represent lung TRM cells that migrate into the airways in response to antigen challenge (55). Together, these findings indicate the importance of lung TRM in protecting against respiratory infections, suggesting that targeting generation of persisting CD4 TRM in the lung would provide optimal protection.

### Reproductive tract mucosal CD4 TRM

The mucosal surfaces of the male and FRT are major sites of entry for sexual transmitted diseases such as herpes simplex virus (HSV), *Neisseria gonorrhoeae*, human papillomavirus, and human immunodeficiency virus (HIV) – all of great public health concern. The reproductive tract is also prone to opportunistic fungal and bacterial infections with increased incidence in immunocompromised (56) and immunosuppressed patients (57), indicating a role for T-cell mediated immunity in preventing these infections. CD4 T cells are thought to be especially important in controlling genital HSV-2 infection, with mouse studies showing that CD8 deficient mice can be successfully vaccinated against disease while CD4 deficient strains are not (58, 59). The importance of CD4 T cells in protection against HSV-2 was supported by the finding that intravaginal HSV-2 infection generates CD4 TRM but little CD8 TRM. These vaginal mucosal memory CD4 T cells in the FRT are sufficient for protective responses to HSV (44) even in the absence of CD8 T cells. In humans, CD4 T cells specific for multiple viral epitopes localize to the uterine cervix (42, 43, 60) and this resident population is thought to limit the severity of recurrent HSV infections (43). As is the case with HSV-2, pre-existing CD4 TRM cells in the RT may be important for conferring protection against other infections of the urogenital tract such as *N. gonorrhoeae, Chlamydia muridarum*, and *Candida* infections (61–64).

The relative contribution of CD4 and CD8 T cells in providing protective immunity in the reproductive tract can vary based on the nature of the invading pathogens; however, new studies indicate that CD4 and CD8 TRM can provide early *in situ* immune responses to infection of the FRT. CD8 TRM have been targeted in the quest to develop a vaccine against HIV because CTLs are thought to be most important for killing virally infected cells. Non-human primate models reveal that the simian immunodeficiency virus (SIV) establishes a small founder population of infected cells in the local tissue after infection (65, 66). This founder population serves as an expanding source of virus that contributes to virus dissemination (66), and presents an opportunity for total elimination of mucosal viral infections during a narrow window of time early after infection. This task may require early *in situ* immune responses mounted by local TRM populations.

### CD4 TRM in the intestines

The intestinal mucosa is a major interface where the body is exposed to environmental antigens, including benign food antigens, beneficial commensal microorganisms as well as dangerous pathogens. Within the intestine are multiple specialized populations of adaptive and innate immune cells that contribute to various immune functions including: oral tolerance to food antigens, tolerance of commensals, and protective immunity against enteric pathogens (67). These populations include memory CD4 T cells, some of which are permanently resident CD4 TRM. Gut T cells are distributed throughout the organized lymphoid tissues that are found throughout the intestines including: Peyers patches, gut-associated lymphoid tissue (GALT), and isolated lymphoid follicles (68, 69). Additionally, gut T cells are also found diffused throughout the lamina propria (LP) and within the intraepithelial (IEL) compartment. The majority of the IEL T cells are CD8^+^ T cells that also express CD103 (70–72) with a lower proportion of CD4 T cells in the IEL compartment. However, CD4 T cells comprise the majority of T cells in the LP and they express an effector-memory phenotype (CD62L^lo^CD44^hi^) (67). In humans, the vast majority of memory CD4 T cells in healthy small and large intestines express CD69, the putative TRM marker (10).

Intestinal resident memory CD4 T-cell populations are shaped by commensal bacterial species. One particular commensal microbe, segmented filamentous bacteria (SFB), was recently shown to induce T_H_17 cells in the LP of mice (73, 74). T_H_17 cells provide mucosal immunity against bacterial pathogens through the production of IL-17 and IL-22 (73, 74). In addition to T_H_17 cells, commensal bacteria induce resident T-cell populations with regulatory function. Studies have shown that a significant proportion of T_regs_ in the intestines are conventional T cells that are converted to a regulatory phenotype in response to the commensal bacterium of the intestinal microbiota (75). Further research revealed specific strains of *Clostridium*, within mouse intestinal commensals, which were sufficient to induce gut resident T_regs_ in mice (76). This group further showed that a selected mixture of *Clostridia* strains from the human microbiota also induced T_regs_ in mice after colonization of the intestines (77). Gut infections with pathogenic bacteria, likewise, induce CD4 TRM populations within the LP. In experimental systems, memory CD4 T cells in the LP are induced by oral infection with bacterial pathogens like *Listeria monocytogenes* (45).

Studies have employed parabiosis and tissue-grafting approaches to show that gut T-cell populations are maintained independently of systemic populations (2, 78, 79). The mucosal immune system of the gastrointestinal tract is a compartmentalized division, including resident memory T-cell populations with both pro- and anti-inflammatory functions, which provide important functions for the physiology of the intestines. It has been shown that gut APCs acquire antigen and migrate to the draining mesenteric lymph nodes where they activate T cells, imprinting the resulting effector and memory T cells to migrate specifically back into the intestines from circulation (80). This migration tropism of gut memory CD4 T cells is similar to that observed with lung CD4 TRM, and may be a distinguishing feature of mucosal CD4 TRM.

## TRM in Chronic Inflammatory Diseases

CD4 TRM have been investigated mainly for their role in providing protective immunity to pathogens that target specific tissues. However, there has been emerging evidence that this population may play a significant role in the pathogenesis of certain autoimmune, allergic, and atopic diseases. In mucosal sites, aberrant immune function and cross-reactivity of CD4 TRM in peripheral tissues are being investigated in inflammatory bowel disease (IBD) and asthma as possible causes of chronic or remitting immunopathology. In addition, there is evidence that CD4 TRM may play deleterious roles in inflammatory disorders of barrier surfaces such as skin. Understanding how CD4 TRM can promote undesirable inflammatory effects in the tissues is important to develop more targeted strategies for therapeutic control of inflammatory diseases.

Allergen-specific TRM populations can be established within lungs following local immune responses induced by exposure to allergens. As is the case following pathogen infection of barrier surfaces, a subset of the effector cells responding to the allergen is imprinted with a TRM phenotype and retained within the tissue. Memory T cells, particularly T_H_2 cells, are strongly involved in the pathogenesis of the chronic manifestations of allergic and atopic diseases (81–83); therefore, their localization at particular tissues make them prone to being reactivated and causing chronic disease. It will be interesting to determine whether CD4 TRM cells are established and maintained within the lung in mouse models of allergic asthma and their role in asthma pathogenesis and also in maintaining the hyper-responsive condition in the tissues. Pathogenic functions of lung CD4 TRM could involve immune cell recruitment into the lung airway upon secondary and chronic allergen exposure.

Inflammatory bowel disease is a chronic inflammatory disease of the gastrointestinal tract characterized by persistent inflammation of the gut or, in some cases, is manifested as a relapsing–remitting syndrome with flare-ups and resolution (84, 85). The chronic recurrence of disease and the restriction of the inflammation to the gastrointestinal tract suggest a role for resident memory T cells in the pathogenesis of IBD. T_H_1 and T_H_17 cells have both been implicated in the pathogenesis of the disease. In experimental models of IBD circulating colitogenic memory CD4 T cells required the presence of gut commensals to induce inflammation and IBD pathogenesis (86). In mouse models of IBD transfer experiments of gut CD4 TRM transferred disease to RAG2^−/−^ mice (87). This demonstrates resident memory CD4 T-cell populations in the gut can propagate local inflammation leading to chronic IBD symptoms.

Psoriasis is another chronic inflammatory disease caused by T-cell responses at a barrier surface (88), with pathogenesis of relevance to inflammation in mucosal sites. Disease pathogenesis is driven by T-cell migration into the epidermis and local production of inflammatory cytokines. T_H_1 and T_H_17 cells in particular have been linked to disease pathogenesis (89, 90). Skin resident memory T cells that are pathological are thought to influence disease recurrence (91). It was recently found that there were elevated numbers of CD8 and CD4 T cells in the dermis of resolved psoriasis lesions. These cells expressed markers associated with TRM (92), including CD103 and the α1β1 integrins expressed on epidermal CD4 T cells (88, 92). It is thus possible that chronic inflammation induces expression of integrins, which mediate cell–cell interactions involved in T-cell retention in the epidermis and establishment of CD4 TRM. The harmful effects of TRM in cases of tissue-specific chronic inflammation, as seen in asthma and psoriasis, make TRM ideal targets for therapeutic interventions.

## Establishment and Maintenance of CD4 TRM

**Figure 1:**
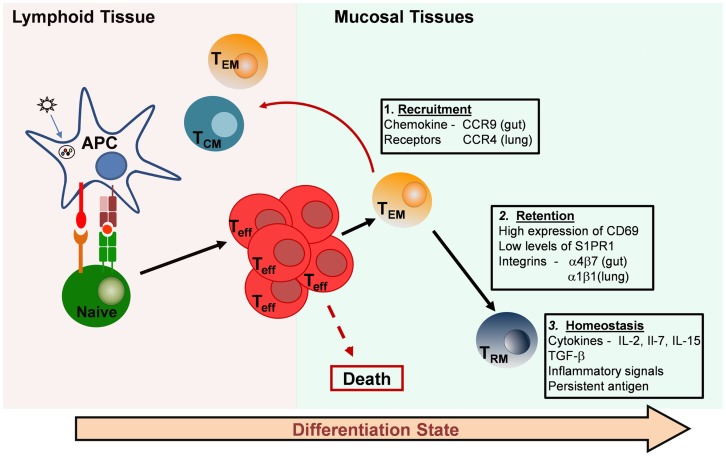
**Generation and maintenance of resident memory T-cell subsets**. Resident memory T cells in mucosal tissues are likely derived from recruited effector T cells that originate in lymphoid organs. Effector cells can be imprinted with specific chemokine receptors that direct migration to individual tissues (Box 1). Most of the effector cells die, but a proportion of the effector or primed T cells differentiates into long-lived resting memory T cells. There are three major types: central-memory T cells (TCM), which migrate back to lymphoid tissue, effector-memory T cells (TEM), which circulate through peripheral tissues and tissue-resident memory T cells (TRM), which are retained in mucosal tissue sites and take up long-term residence there without recirculating. Retention of TRM in peripheral tissues is thought to be mediated by the inhibition of egress through S1PR1 and by cell–cell interactions facilitated by integrin expression (Box 2). Maintenance and homeostasis of TRM in mucosal tissues may depend on pro-survival cytokines, constitutive low-level inflammation, and the persistence of antigen at the site (Box 3).

The factors involved in the establishment and maintenance of TRM populations within non-lymphoid tissues are not clearly understood. Entry of effector T cells into non-lymphoid mucosal sites is controlled by the expression of certain chemokine receptors, selectins, and integrins, which are universally upregulated after T-cell activation, regardless of the secondary lymphoid tissue where cells were activated (93). It has also been shown, however, that T cells primed by dendritic cells in certain lymphoid sites are programed to home specifically to certain tissues (94, 95). This tissue-specific homing is mediated through the expression of various integrins and chemokine receptors, which are involved in cell migration into specific tissues. For example, chemokine receptor chemokine receptor 9 (CCR9) and integrin α4β7 target T cells to the intestines (96, 97), cutaneous leukocyte antigen (CLA) targets cells to the skin (98), and lung DC promote effector T-cell homing to the lung through upregulation of CCR4 (99) (Figure 1). Whether these specific chemokine receptors persist in TRM remains to be established, and identifying specific tissue signatures for TRM in distinct sites is an active area of study in the field.

Effector T cells responding to infection/inflammation within non-lymphoid or mucosal tissues may further respond to inflammatory and/or tissue-specific environmental factors, which impart them with a resident memory phenotype. For CD8 T cells, expression of transforming growth factor-beta (TGF-β) within certain tissues (100, 101) induces the expression of the mucosal integrin, αE(CD103)β7 (102, 103), which is responsible for retention of CD8 TRM in non-lymphoid tissues. CD4 TRM at mucosal sites express CD103 at a much lower frequency compared to CD8 TRM (10, 12) and may be maintained by other, unknown mechanisms. Other integrins may be involved in CD4 TRM retention and residence, which may represent a major difference between CD4 and CD8 TRM in the same tissue. It has been found that the vast majority of CD4 T cells persisting in the lung airways following influenza virus infection express the α1β1 integrin (VLA-1) while virus-specific cells in lymphoid sites have low expression of VLA-1 (37). Secondary infection with IAV revealed that these VLA-1^+^ cells represented 80% of the early producers of IFN-γ (37) suggesting that the α1β1 integrin might be a marker of lung CD4 TRM cells. CD11a or LFA-1 is also expressed at higher levels in lung CD4 TRM compared to circulating CD4 TEM (12, 13), and may also contribute to tissue retention.

The lectin CD69 is constitutively expressed on CD4 and CD8 TRM in all the tissues that have been described (3, 13, 78, 104). Traditionally, CD69 has been thought of as an early activation marker of T cells, being transiently upregulated early after activation through the T-cell receptor (105) or in response to proinflammatory cytokines, including type I interferons (IFN-α and IFN-β) and tumor necrosis factor-α (106, 107). TRM cells in the lung constitutively express elevated levels of CD69 while T cells of the same specificities express low levels of CD69 in the lymph node and spleen (12). This local expression of CD69 by TRM may be the result of continued stimulation through encounters with persistent antigen at tissue sites, which has been observed following influenza virus (108, 109). We found that acquisition of TRM properties by effector cells adoptively transferred into congenic hosts in a manner that is independent of antigen (13). Induction of CD69 expression by T cells within tissues may therefore be the result of the environmental milieu associated with mucosal tissues, which is likely to be quite different from that of lymphoid organs.

Tonic signaling, through low levels of cytokines produced in response to environmental antigens, may also be involved in the differentiation of effector and effector-memory T cells into TRM. CD69 is thought to play a functional role in T-cell retention within tissues because of its regulation of sphingosine-1-phosphate receptor 1 (S1PR1) (110), which play a role in the egress of lymphocytes from certain tissues (111). A summary of processes involved in the recruitment, retention, and homeostasis of TRM in peripheral tissues is provided in Figure 1. Further studies are needed to define the exact molecular determinants of CD4 TRM establishment and maintenance. Defining the differences and similarities between the requirements for CD4 and CD8 TRM development and maintenance in tissues is also of utmost importance for the targeting of these new subsets by vaccines and therapeutics.

## Implications for Vaccines

As outlined in Table 1, there is now evidence for the presence CD4 TRM in multiple mucosal sites and roles for this subset in protection against pathogenic infections (Table 1). These findings present important implications for future therapeutic developments for promoting protective responses *in situ*. In the lung, generation of TRM populations targeting respiratory pathogens may significantly reduce the mortality and morbidity associated with these infections. In the case of influenza virus, the more common subunit vaccine is administered by intramuscular immunization, while the live attenuated influenza virus vaccine (LIAV), which is more commonly used for younger individuals, is administered intranasally. Both types of vaccine have been optimized for the generation of protective antibodies; however, both vaccines can induce circulating virus-specific T cells (112, 113) with the LIAV vaccine thought to generate more tissue-tropic T cells (114). A vaccine that induces memory T cells that recognize conserved epitopes from internal viral proteins could form the basis of a universal influenza virus vaccine. It may also be important that such a vaccine is administered in a manner that generates protective memory T-cell populations resident in the lung for optimal protection, likely via the intranasal route.

In the case of Mtb, current intramuscular bacille Calmette–Guérin (BCG) vaccination protocols show reliable protection during childhood but protection wanes during adulthood (115). This protection is mediated by T_H_1 memory cells; however, the exact effector mechanisms by which Th1 memory protect is not fully understood. Recent attempts to boost BCG protection by parenteral vaccination have yielded disappointing results. For example, clinical trials of the recombinant vaccinia virus booster vaccine, MVA85A, did not show better efficacy than the BCG vaccine (116) even though the new vaccine generates highly durable Mtb-specific T_H_1 responses (117). This result may have been foreshadowed by mouse experiments showing that parenteral boost with MVA85A after BCG priming showed no improvement in protection (118–121), compared with BCG vaccination alone, even with each vaccine showing high immunogenicity. Improved protection over BCG alone is only observed after multiple immunizations, which induce entry of cells into non-lymphoid tissues (122). These results suggest that memory T-cell mediated protection against respiratory Mtb infection may depend on the early *in situ* effector functions of TRM populations. Optimal protection may require both parenteral and mucosal administration of vaccines, which will generate both TRM and lymphoid memory populations.

The prevalence and protective capacities of TRM in the FRT has encouraged efforts for generating *in situ* vaccines for protection against sexually transmitted diseases. A new strategy for generating TRM in the FRT involves a “prime and pull” technique in which parenteral vaccination (prime) is combined with recruitment of activated T cells into the genital tract by local application of a chemokine (pull). When applied to the mouse HSV-2 infection model, this approach resulted in the recruitment but not retention of CD4 memory T cells, although HSV-2-specific CD8 TRM were generated (7). These results suggest that the establishment of CD4 TRM in the reproductive tract may require additional signals, such as those present during HSV infection (44, 123, 124). In other studies for HIV vaccines, intranasal vaccination was found to generate higher anti-SIV T-cell responses in the colorectal mucosa, increased numbers of gut-tropic α4β7 cells in circulation, and a longer disease-free period compared to vaccination via the intramuscular route (125). These findings suggest some connections between mucosal sites important for assessing the optimal route of administration, and perhaps suggesting that a pull step may not be necessary. Further studies are needed to define the signals necessary for the local differentiation of CD4 T cells into TRM in order to develop vaccination and therapeutic protocols that harness the unique properties of these cells to prevent and fight site-specific infections.

## Concluding Remarks

Compartmentalization of immunological memory in diverse non-lymphoid and mucosal tissues may be a central mechanism underlying the long-term persistence and efficacy of T-cell memory to systemic and site-specific pathogens. CD4 TRM in mucosal tissues may be optimally poised to orchestrate the immune response to recurring tissue-tropic infections. Developing vaccines that therefore generate this important population in targeted tissues should be a major focus of future research; however, greater understanding of the mechanisms involved in imprinting tissue-resident CD4 T cells is needed. Elucidating strategies to target TRM in mucosal and tissues will also allow for the development of therapeutics that reduce TRM populations in various tissues in instances of aberrant immune responses and immunopathology.

## Conflict of Interest Statement

The authors declare that the research was conducted in the absence of any commercial or financial relationships that could be construed as a potential conflict of interest.
